# Supplementary material for: Functional, Physical, and Volatile Characterization of Chitosan/Starch Food Films Functionalized with Mango Leaf Extract
Source: Foods. 2023 Aug 7;12(15):2977. doi: 10.3390/foods12152977 (PMC10418412; doi:10.3390/foods12152977)
Supplement: Supplementary file 1 [file foods-12-02977-s001.zip › foods-2543266-supplementary.pdf]

# Functional, Physical, and Volatile Characterization of Chitosan/Starch Food Films Functionalized with Mango Leave Extract

Cejudo Bastante, C.[1], Ferreiro González, M.[2], Romera González I.[1], Casas Cardoso, L.[1], Mantell Serrano, C[1].

SUPLEMENTARY MATERIAL

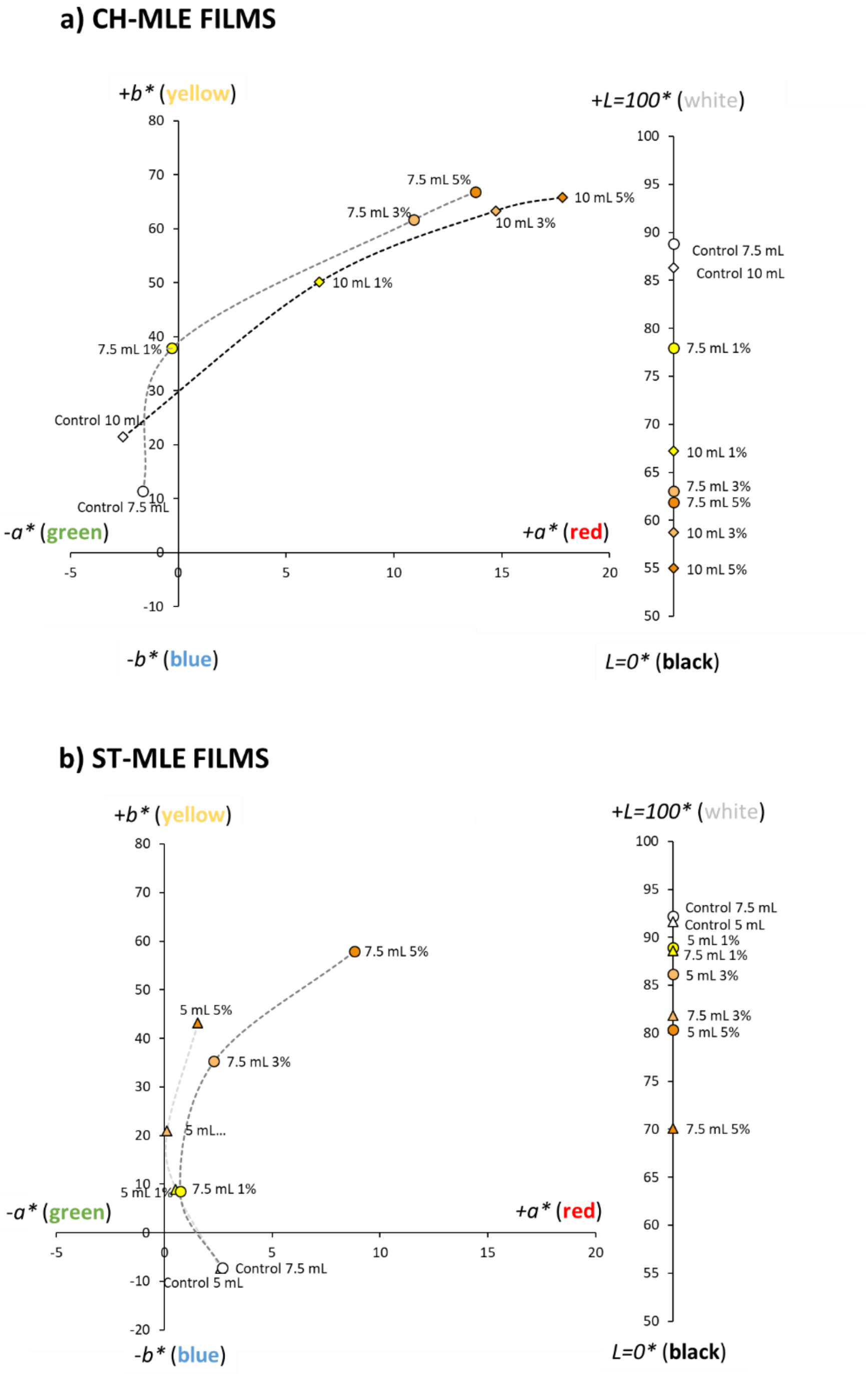


**Figure S1.** CIELAB coordinates of CH (A) and ST (B) films manufactured using different volumes (Δ:5 mL;○:7.5 mL;◊:10 mL) and MLE percentages.
